# Structural Studies of Aluminated form of Zeolites—EXAFS and XRD Experiment, STEM Micrography, and DFT Modelling

**DOI:** 10.3390/molecules26123566

**Published:** 2021-06-10

**Authors:** Gabriela Jajko, Paweł Kozyra, Maciej Strzempek, Paulina Indyka, Marcin Zając, Stefan Witkowski, Witold Piskorz

**Affiliations:** 1 Faculty of Chemistry, Jagiellonian University, ul. Gronostajowa 2, 30-387 Kraków, Poland; gabriela.jajko@doctoral.uj.edu.pl (G.J.); kozyra@chemia.uj.edu.pl (P.K.); strzempek@chemia.uj.edu.pl (M.S.); paulina.indyka@uj.edu.pl (P.I.); witkowss@chemia.uj.edu.pl (S.W.); 2 Małopolska Centre of Biotechnology, ul. Gronostajowa 7A, 30-387 Kraków, Poland; 3 National Synchrotron Radiation Centre SOLARIS, Jagiellonian University, ul. Czerwone Maki 98, 30-392 Kraków, Poland; mar.zajac@uj.edu.pl

**Keywords:** zeolites, EXAFS, DFT, HR-TEM, XRD

## Abstract

In this article, the results of computational structural studies on Al-containing zeolites, via periodic DFT + D modelling and FDM (Finite Difference Method) to solve the Schrödinger equation (FDMNES) for XAS simulations, corroborated by EXAFS (Extended X-ray Absorption Fine Structure) spectroscopy and PXRD (powder X-ray diffractometry), are presented. The applicability of Radial Distribution Function (RDF) to screen out the postulated zeolite structure is also discussed. The structural conclusions are further verified by HR-TEM imaging.

## 1. Introduction

This work is aimed at the structural analysis of the zeolites used in our previous studies [1] to support consideration of the Al distribution. EXAFS spectroscopy was used due to its specific properties, discussed below.

### 1.1. Importance of Zeolites

Zeolites belong to the most important group of large-scale catalysts in the petrochemical industry and also serve as molecular sieves, sorbents, or ion exchange systems [2,3,4]. They have also found application in the hydrocarbon field, namely, in the production of olefins from non-conventional, non-oil sources such as natural gas, coal, and biomass [5,6].

### 1.2. Zeolite Structure

According to the common classification [7], zeolites belong to the family of (micro)porous tectosilicates. The 3D structure of the siliceous zeolites is built up of corner-sharing primary building units (PBU), the ${\left[{\mathrm{SiO}}_{4}\right]}^{4-}$ tetrahedra, which can be substituted by foreign sites, most frequently ${\left[{\mathrm{AlO}}_{4}\right]}^{5-}$ units. Such a modification introduces the negative charge to the framework which is necessarily neutralised by the off-lattice cations, e.g., protons. The other cations, counterbalancing the lattice charge, can be the *n*-valence $M^{n+}$ cations, shared by *n*${\left[{\mathrm{AlO}}_{4}\right]}^{5-}$ units. The electrostatic repulsion between the formally negatively charged ${\left[{\mathrm{AlO}}_{4}\right]}^{5-}$ tetrahedra prevents them from occupying the adjacent position. The necessity of their separation by the ${\left[{\mathrm{SiO}}_{4}\right]}^{4-}$ units is called the Löwenstein rule [8] (‘aluminium avoidance’) and its less restrictive, and more recent, formulation also based on the electrostatic repulsion of negative charges ${\left[{\mathrm{AlO}}_{4}\right]}^{5-}$, is called the Dempsey rule [9]. These rules are not, however, absolute and zeolites with Al–O–Al chains do exist [10].

The other cations, e.g., the TM cations, responsible for the redox properties of the zeolitic systems, can also be introduced into the zeolite framework, and this property is most often accompanied by the presence of acidic centres, thus forming the bi-functional catalysts [11]. The TM ions can occupy both the framework positions (via the isomorphic substitution), see, e.g., [12,13,14,15], or the extraframework positions (ion exchangeable sites) [16,17,18]. Due to easier accessibility, the latter case is most commonly found in catalysis. Zeolites can accommodate not only the bare cations [19] or small clusters (e.g., mono-(μ-oxo)dicupric [20]), but also complexes such as Ru(III) benzimidazole or 2-ethyl Ru(III) benzimidazole [21].

**Figure 1:**
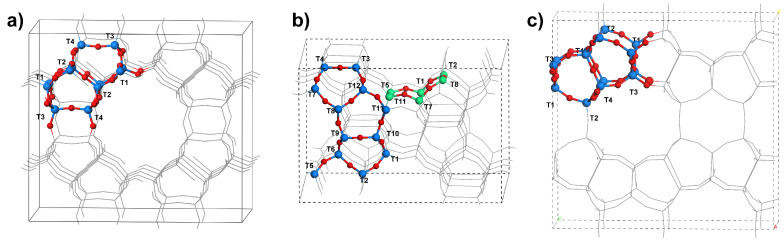
Annotated Si positions in framework of (**a**) MOR, (**b**) MFI, (**c**) FER; blue spheres—the non-equivalent Si positions, red spheres—O atoms, green spheres—commonly regarded set of Si atoms exposed to large channels. After [11], copyright by Springer International Publishing, with permission.

The non-equivalent (in siliceous zeolites) classes of *T* sites (standardised [22]) are presented in Figure 1. In siliceous chabazite, all *T* sites are equivalent.

### 1.3. Localisation of Aluminium Centres in Zeolite Structures

The isomorphous substitution of ${\left[{\mathrm{SiO}}_{4}\right]}^{4-}$ by ${\left[{\mathrm{AlO}}_{4}\right]}^{5-}$, leading to the introduction of the ${\mathrm{Al}}^{3+}$ cations in the *T*-sites (tetrahedral sites) of zeolites, yields the formation of the ion-exchange and Brønsted/Lewis acidic sites. Hence, it is also crucial for the catalytic properties of the modified zeolites [23,24]. The localisation and coordination of the Al sites in zeolites are essential for the understanding of the sorption properties of these materials and are also one of the key factors determining acidity and, as a consequence, their catalytic activity. The presence and the chemical environment of the Al sites in the zeolite framework determine the abundance and activity of the Lewis/Brønsted acidic sites, decisive for C–C bond making or breaking and thus determining the activity and selectivity of the resultant substituted zeolite catalyst.

The presence and concentration of aluminium sites in the zeolite framework can be assessed by many methods, e.g., elemental analysis. The issue of the local structure (the position of the ${\left[{\mathrm{AlO}}_{4}\right]}^{5-}$ units in the zeolite framework) has been, however, a matter of miscellaneous studies, both computational [17,25,26,27] and experimental [25,28,29,30,31,32,33,34,35], for decades. It is also known that the localisation of the Al sites is strongly dependent on the synthesis procedure and hard to reproduce [36,37]. This issue is important from both a fundamental and practical point of view.

### 1.4. Extraframework Cations

As was mentioned in the section ‘*Zeolite Structure*’ (*vide supra*), besides protons, other cations can also be introduced into the zeolite framework, adapting an extraframework position.

The TM-zeolites are active in plethora of processes, e.g., the direct oxidation of ${\mathrm{CH}}_{4}$ to ${\mathrm{CH}}_{3}\mathrm{OH}$ [38], nitrogen oxide decomposition [39,40], and H–H bond activation [19,26,41]. For the partial methane oxidation a number of zeolitic systems were studied, e.g., Fe/ZSM-5 [42,43], Cu/ZSM-5, FeCu/ZSM-5 [44], Fe/FER [45], and Cu/MOR [20]. The TM-zeolites systems are also active in the alkane/arene like dehydrogenation [46], aromatization of light alkanes [47,48], and also in alkylation [49] and selective oxidation [50] processes. In particular, in the deNOx process, the transition metals: Cu [51,52,53,54,55], Fe [56], V [57], Co [38], Ni [38], etc. in zeolites are the common catalysts.

### 1.5. EXAFS

From the X-ray spectroscopy family, EXAFS is the spectroscopy that analyses the part of the X-ray absorption spectra between ca. +50 to +1000 eV above the absorption edge energy [58]. This spectroscopy has its root in the features which can be semi-quantitatively described by the single scattering of the excited electron. The complementary XAS spectroscopy, XANES, is based on the multiple scattering of the excited electron and can thus provide information on higher correlations, e.g., the bond angles. It is, however, more difficult for interpretation.

It was found [59] that some information on Al distribution can be extracted by conducting EXAFS modelling and experiments and, thus, the characteristics of the local environment of selected atoms is the state-of-the-art of this technique [60,61,62]. It was shown that ^27^Al MAS NMR is problematic in non-standard conditions due to the quadrupolar nature of the aluminium nucleus [59]. The EXAFS spectroscopy is element-selective and does not require long-range ordering of the structure, contrary to diffraction methods [58]. The strength of EXAFS is also visible when the (crystalline) structure has some substitutional disorder, e.g., in Al-exchanged zeolites when the exchange of *T*-sites is not regular enough to be studied by diffraction methods. In such cases, the elemental selectivity, caused by the ability of EXAFS to probe the environment of atoms of the selected element only, is a significant advantage. In spite of non-periodic Al distribution, Al atoms located in non-equivalent positions, due to their different environment, contribute to the signal χ(r) with different components. Fortunately, the components can be obtained by modelling and used for interpretation of the results.

The caveat of the EXAFS study of aluminated zeolites is the closeness of the absorption edges of Al and Si, which limits the extent of acquisition of the spectra (*vide infra*). It also reveals the advantages of quantum–chemical modelling for the determination of the Al site occupancy.

## 2. Experimental Part

### 2.1. Materials

The mordenite (MOR), ferrierite (FER), and ZSM-5 (MFI) samples of various Si/Al ratio and of the average crystal diameter of ca. 200 nm, were used for all investigations. The zeolites were provided in ammonium–exchanged form and converted to the protonic form by heating in air at 550 °C. A detailed description of the used samples is given in Table 1.

### 2.2. Computational Models

**Figure 2:**
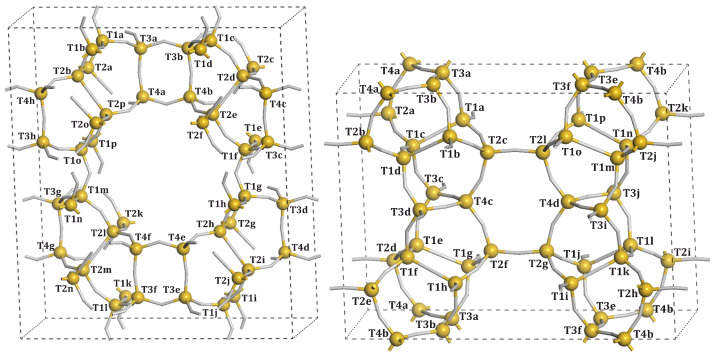
MOR (**left structure**) and FER (**right structure**) unit cell with labelled *T* sites.

**Figure 3:**
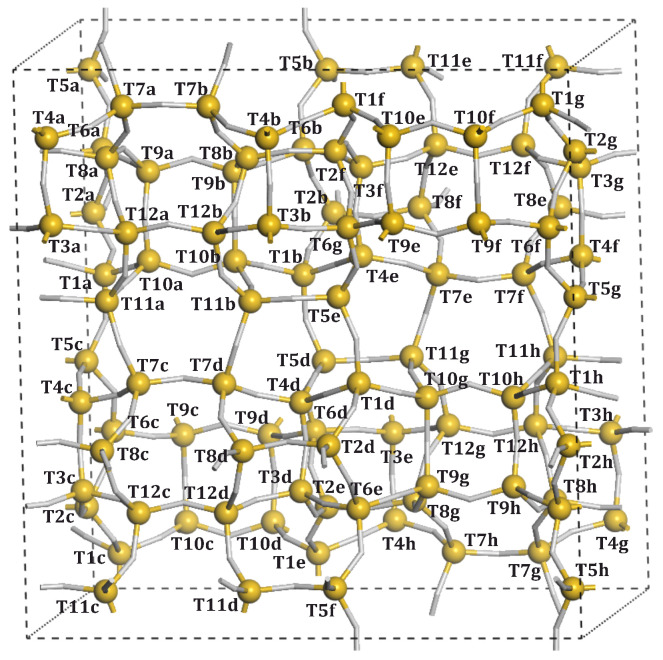
MFI unit cell with labelled *T* sites.

The T*n* sites, available for Al substitution, are presented in Figure 2 and Figure 3. Due to the symmetry lowering imposed by Al substitution, they are distinguished by the letters appended to the T*n* labels. The particular substitutions for the specific model numbers are presented in Table 2.

### 2.3. X-ray Spectroscopy

The sequence of the structures was studied employing EXAFS at the 04BM beamline at the National Synchrotron Radiation Centre SOLARIS [63]. The total electron yield (TEY) mode was used allowing for a depth of sampling of a few nanometres. The spectra were acquired at the room temperature. The powder samples were pressed in the carbon film (compatible with UHV) and attached to the standard titanium flag-style Omicron holders for assuring their conductivity and thus protecting them from the X-ray induced charging. The X-ray energy was calibrated using an Al foil, setting the energy at the first maximum of the first derivative to 1560 eV. The beamline optics with plane grating monochromator allow for measurements with an energy resolution of 400 meV with the moderated flux (10^9^ ph/s).

### 2.4. XRD

The diffractograms were obtained with the Rigaku Miniflex 600 X-ray diffractometer equipped with a DeTEX detector, using Cu Kα radiation (λ=1.540598 Å), recorded in the range of 2Θ = 10—70° with steps of 0.02°/s. Rietveld analysis was performed with use of the MAUD code [64,65].

### 2.5. TEM Microscopic Studies

Transmission Electron Microscope (TEM) imaging was performed with use of FEI Tecnai Osiris microscope with an X-FEG Schottky-type field emission gun at 200 kV accelerating voltage. The morphology of the synthesised zeolites was assessed by Scanning Transmission Electron Microscopy (STEM) measurements. The microscope was equipped with a High Angle Annular Dark Field (HAADF) detector (Fischione 3000). The camera length was kept in the range 330–550 mm to maximise the HAADF signal intensity. Samples for electron microscopic characterisation were deposited on a lacey carbon-coated film supported on a Cu TEM grid (Agar Scientific, 300 mesh).

### 2.6. Computational Details

All the quantum–chemical calculations of energies and geometries were performed at the DFT level of theory with the use of the VASP [66,67] code, version 5.4.4, which utilises the PAW [68,69] method to reconstruct the all-electron wave function. Gaussian smearing (preferred over the Methfessel–Paxton smearing [70] for the description of insulators) with σ=0.01 eV was used. The following optimisation criteria were used: energy change of $10^{-6}$ eV between two successive steps for the SCF, gradient norm of $10^{-3}$ eV/Å for the geometry optimisation. A basis set plane-wave cutoff energy of 500 eV and the PBE [71,72] (from the GGA family) correlation–exchange functional was used. The dispersion interactions were accounted for using the Grimme semiempirical method [73]. The cell lattice constant was optimised by fitting E/V to the Birch–Murnaghan equation of state [74].

For the simulation of XAS spectra (in particular, the EXAFS spectra) a very general and successful method for solving the differential equation, the Finite Difference Method (FDM) method, was used to solve the Schrödinger equation for modelling the XAS spectra, which allows avoiding the often used crude muffin-tin approximation of the electrostatic potential. Such procedure is well-known for its high accuracy in the reproduction of, e.g., Al K-edge EXAFS spectra [75,76], superior to the accuracy of the muffin-tin-based approach. The code used for the EXAFS simulations was FDMNES [77,78,79], which allows for the selection between two levels of scattering theory, described above.

The spectra were simulated for the energy range of −25 to 300 eV, relative to the absorption edge, with a step of 0.5 eV. The scattering radius of 12.0 Å was used, and all multiple scatterings within it were accounted for. The relativistic effects were taken into account.

The resultant μ(E) values were further processed with the use of the Athena code (from the Ravel’s famous ‘*six-pack*’ [80,81]) to normalise the spectra and to eventually obtain the χ(r) function. For the χ(k) signal apodisation, the Hanning window function was used.

## 3. Results

The total of 33 structures (see Table 2) of MOR (16 structures), FER (1), and ZSM-5 (16), with the Si/Al ratio close to that experimental, and with different Al site localisation was designed and their structures were optimised at the DFT level of theory. In all cases, the 1×1×1 computational unit cell was used. During the procedure of constructing the models, the number and differentiability of particular *T* sites was taken into account as well as that the Löwenstein rule was preserved.

### 3.1. XRD

**Figure 4:**
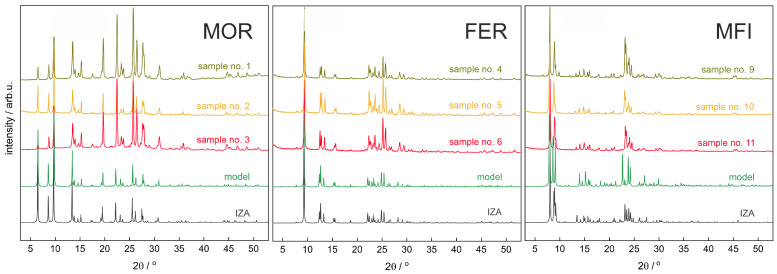
PXRD diffractograms of silicalite (IZA), studied samples, and computational models for MFI, MOR, and FER.

A series of PXRD diffractograms have been registered for the zeolite samples (Table 1). In juxtaposition with the simulated diffractograms (MAUD code), obtained for the structures from the Database Zeolite Atlas (IZA) and for the computational models (Figure 4), the assumptive structure of studied samples (FER, MFI, MOR) was confirmed. The analysis of the differential diffractograms (Figure S1 in SI) also shows that the diffractograms of computational models (labelled ‘model’ in the figure), also with silicon atoms substituted by aluminium atoms, are almost identical compared to those generated for the silicalite structures (for MOR and FER). Thus it means that the diffractograms are not sensitive enough to the particular aluminium atom distribution (even if the effect of aluminium presence is translationally propagated). Moreover, it indicates that the DFT optimisation gave almost undistorted geometry for FER and MOR. In the case of MFI, the diffractograms generated for the structure from database (IZA) and for model of silicalite exhibit only minor differences, shown in Figure S2 in Supporting Information—a (101) reflection shift at 2θ around 8.0°, and as a consequence for the (051) reflection at 2θ= 23.08°). This difference for models containing Al atoms is, however, slightly more evident in the case of MFI, for which the diffractograms for all models with Al atoms do not differ noticeably. The diffractograms registered for the samples provide evidence of good crystallinity and differ from the generated ones only in intensities, FWHM, and background (see SI, Figure S1). The experimental results also allowed for the estimation of the crystal size and the size distribution.

The average nanocrystalline size was calculated from the line width at half maximum intensity (FWHM), with use of the Debye–Scherrer formula:

$$D_{\mathrm{aver}}=\langle \frac{K\lambda }{\beta \cos\Theta }\rangle ,$$

where $D_{\mathrm{aver}}$ is the average crystalline size; *K* is the dimensionless shape factor (the Scherrer constant; here 0.9 ); λ is the wavelength of the used Cu $K\alpha_{1}$ radiation, λ=1.540598 Å; β is the line broadening after subtracting the instrumental line broadening; Θ is the Bragg angle. The instrumental broadening was accounted for by the acquisition of the standard Si powder data recorded under the same conditions.

Due to the plethora of possible miscellaneous defects in the relatively complex structure of zeolites, the issue of the imperfections of the zeolite grains crystallinity was studied *via* the broadening of the diffraction peaks, analysed by the Williamson–Hall regression procedure, described, e.g., in a very comprehensible way in Ref. [82]. The uniform deformation stress model (UDSM) was assumed. Accordingly,

$$\beta \cos\Theta =\frac{K\lambda }{D_{\mathrm{aver}}}+4\sin\Theta \cdot \varepsilon_{\mathrm{UDSM}},$$

where ε is the relative strain. The results are summarised in Table 3.

### 3.2. Pre-Screening of the Potential Zeolite Structures via RDF

Because the computational cost of the numerous XAS computational jobs is relatively high, a pre-screening procedure was undertaken for each structure, namely the Radial Distribution Function (RDF) was calculated and analysed. The RDF was calculated for all the pairs constituted by each of the Al atoms (the first element of a pair) and the atom among all the other atoms within the radius of 10.0 Å (the second element of the pair). The RDF calculations were performed with use of RASPA [83,84] code. The obtained histograms were compared to the experimental χ(r) function. In certain cases, the RDF analysis allowed for attribution of the model spectrum to the experimental χ(r). Particularly, in the computational spectra, the peaks stemming from the H atoms (such attribution was made based on the RDF analysis) are observed although are very weak (the lowest atomic number of H). They do not, however, contribute to the experimental spectra, due to both low atomic number and delocalisation of the very mobile H atoms. The mobility effect (Debye–Waller factor) of the atoms was not included in the simulations (the dynamic matrix was not computed). Such feature of the H-peaks leads to their exclusion from the assessment of the fitness of the modelled spectra to the experimental ones (see SI Figure S3). Moreover, the RDF analysis allowed for the subsequent determination of the distances between Al–Al pairs in the structures.

### 3.3. EXAFS

Since the Al distribution in zeolites is a case of sparse distribution of emitting atoms in a low symmetry (and hence rather complicated, with many variable structural parameters) local environment, the simplified, qualitative EXAFS analysis seems applicable for our purpose. Namely, the positions of a first few shells, corroborated by the RDF analysis, should allow for the assignment of Al to the proper $T_{n}$ site by matching the modelled spectra to the experimental ones. The complexity of the pair distribution function (*vide infra*) supports this reasoning. It was also assumed that, derived from the above mentioned, the distances between Al and O in the ${\left[{\mathrm{AlO}}_{4}\right]}^{5-}$ tetrahedra are identical.

**Figure 5:**
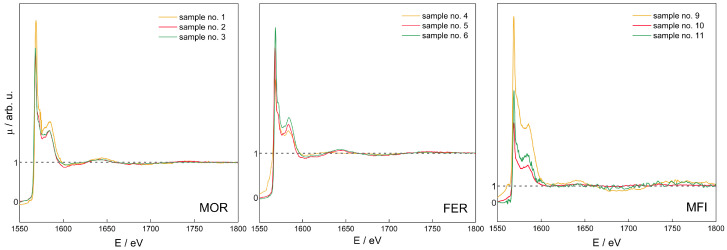
Normalised μ(E) spectra for MOR (Si/Al = 15, 10, and 8 for samples 1 *, 2, 3, respectively; **left**), FER (Si/Al = 9 for samples 4, 5, 6, resp.; **middle**), and MFI (Si/Al = 11.5, 25, 40 for samples 9, 10, 11, resp.; **right**). * see Table 2 for sample numbering.

A series of XAS μ(E) spectra for all samples has been recorded, see Figure 5, and in order to find the proper Al site distribution, Fourier transform (FT) analysis of μ(E) was performed to obtain χ(r). The latter spectra can be regarded as the “pseudo-radial distribution function” [85]. The absolute value of the complex spectra, $\left|\chi (r)\right|$, is discussed further in this text.

For the calculated EXAFS χ(r) spectra, the coefficient for root of mean squares (RMS), $f_{\mathrm{RMS}}$, was calculated as a measure of the experimental results fitness among matching pairs of lines ($r_{i}$ values, obtained *via* the spectra deconvolution):

(1) $$f_{\mathrm{RMS}}=\sqrt{\frac{\sum_{i=2}^{n}{\left(r_{i}^{\exp}-r_{i}^{\mathrm{calc}}\right)}^{2}}{n}},$$

where $r_{i}^{\exp}$ and $r_{i}^{\mathrm{calc}}$ denote the relative distance of the *i*-th shell with respect to the first one (the first Al–O shell in the ca. 1.8 Å range); for the experimental and calculated results, *n* equals 4 or 5, respectively. The modelled spectra were superimposed over the experimental ones in a way so that the first maxima (Al–O shell) thereof are aligned (hence the summing in Equation (1) starts with 2). Such RMS analysis for different pairs Al–*X* ($X\in \left\{\mathrm{Si},\;\mathrm{Al},\;O,\;H\right\}$) allows for the assignment of the shells to the *X* element.

**Figure 6:**
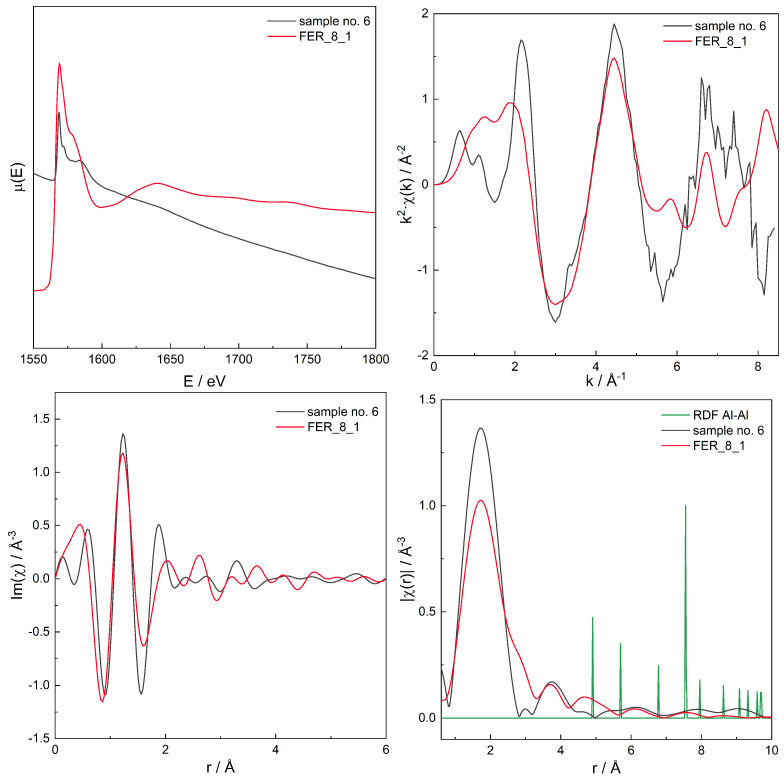
Spectrum of μ(E) (**upper left panel**), $k^{2}\chi \left(k\right)$ (**upper right**), ℑχ(r) (**lower left**), and $\left|\chi (r)\right|$ (**lower right**) for sample no. 6 * (FER of Si/Al = 9), and simulation for computational model labelled FER_8_1, augmented with the Al–Al RDF. Matching RMS = 0.0443. The simulation model characterised by Al–Al distances of: 4.9, 5.7, 7.5, and 9.5 Å. * see Table 2 for sample numbering.

**Figure 7:**
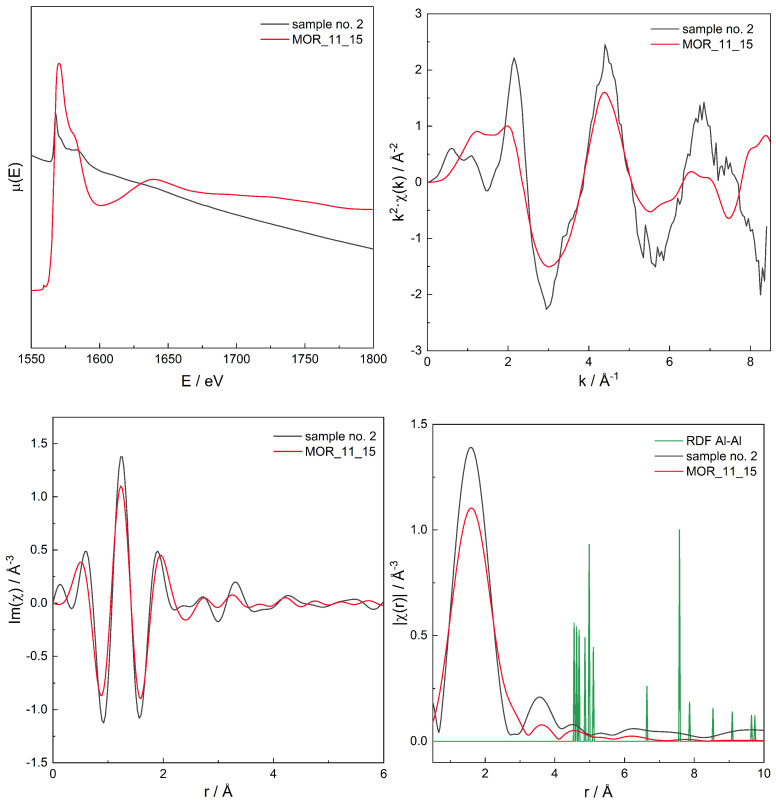
Spectrum of μ(E) (**upper left panel**), $k^{2}\chi \left(k\right)$ (**upper right**), ℑχ(r) (**lower left**), and $\left|\chi (r)\right|$ (**lower right**) for sample no. 2 (MOR of Si/Al = 10) and simulation for computational model labelled MOR_11_15. Matching RMS = 0.0497. The simulation model characterised by Al–Al distances of: 4.5, 5.0, 7.5, and 9.1 Å.

**Figure 8:**
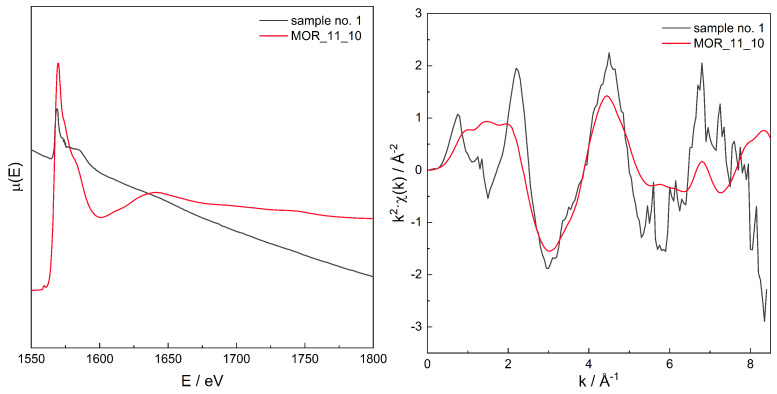

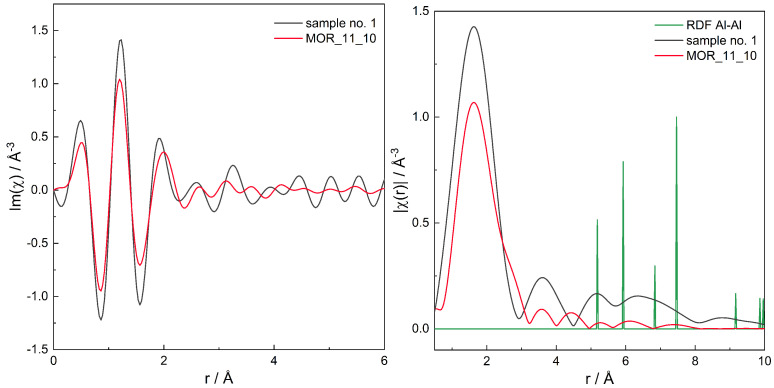
Spectrum of μ(E) (**upper left panel**), $k^{2}\chi \left(k\right)$ (**upper right**), ℑχ(r) (**lower left**), and $\left|\chi (r)\right|$ (**lower right**) for sample no. 1 (MOR of Si/Al = 15) and simulation for computational model labelled MOR_11_10. Matching RMS = 0.1445. The simulation model characterised by Al–Al distances of: 5.2, 6.0, 7.5, and 9.9 Å.

**Figure 9:**
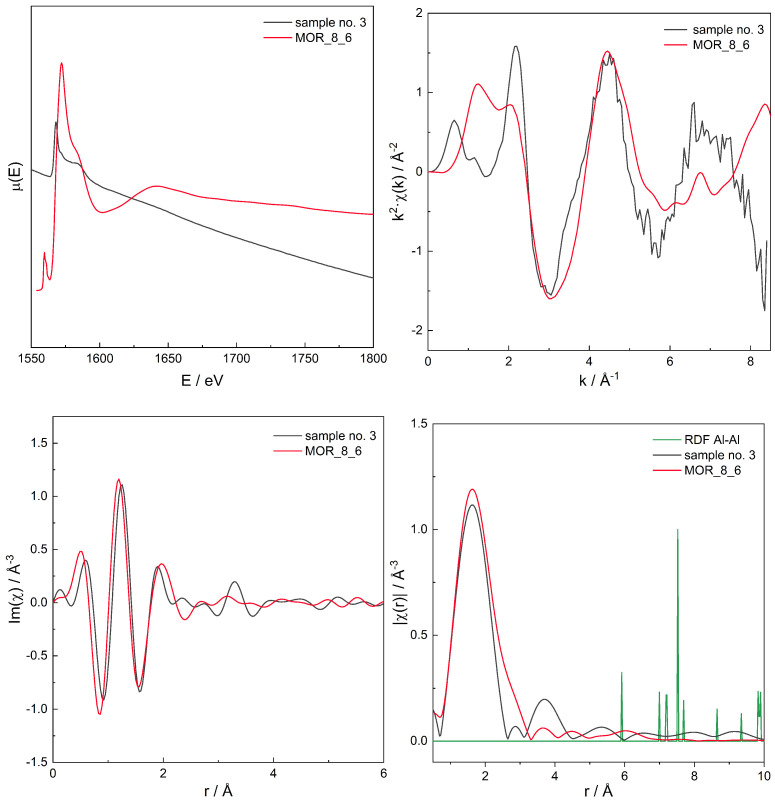
Spectrum of μ(E) (**upper left panel**), $k^{2}\chi \left(k\right)$ (**upper right**), ℑχ(r) (**lower left**), and $\left|\chi (r)\right|$ (**lower right**) for sample no. 3 (MOR of Si/Al = 8) and simulation for computational model labelled MOR_8_6. Matching RMS = 0.0529. The simulation model characterised by Al–Al distances of: 5.9, 7.0, 7.5, 8.5, and 9.9 Å.

**Figure 10:**
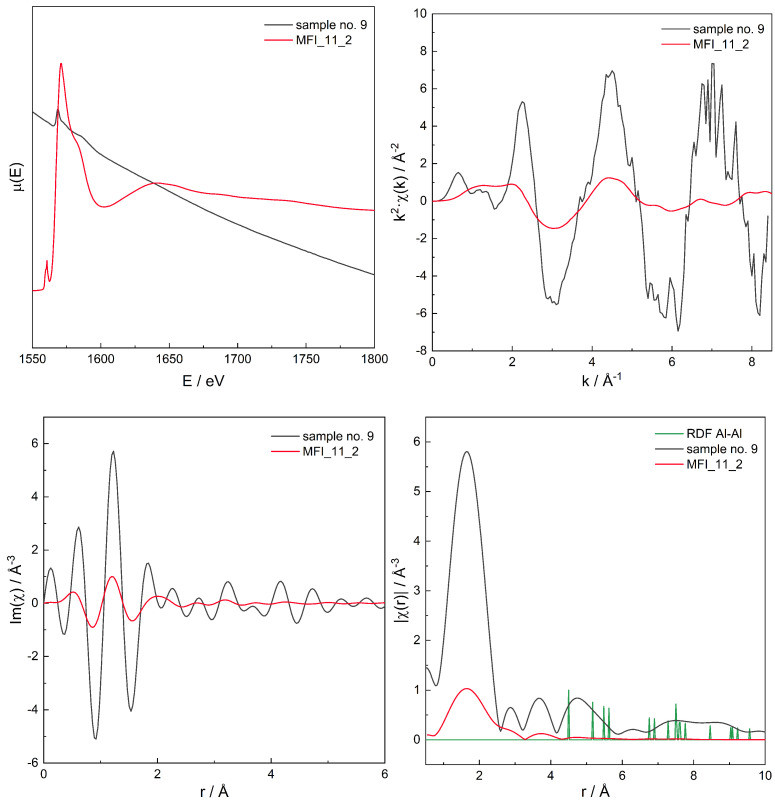
Spectrum of μ(E) (**upper left panel**), $k^{2}\chi \left(k\right)$ (**upper right**), ℑχ(r) (**lower left**), and $\left|\chi (r)\right|$ (**lower right**) for sample no. 9 (MFI of Si/Al = 11) and simulation for computational model labelled MFI_11_2. Matching RMS = 0.0658. The simulation model characterised by Al–Al distances of: 4.6, 5.5, 7.2, 9.3, and 10.4 Å.

**Figure 11:**
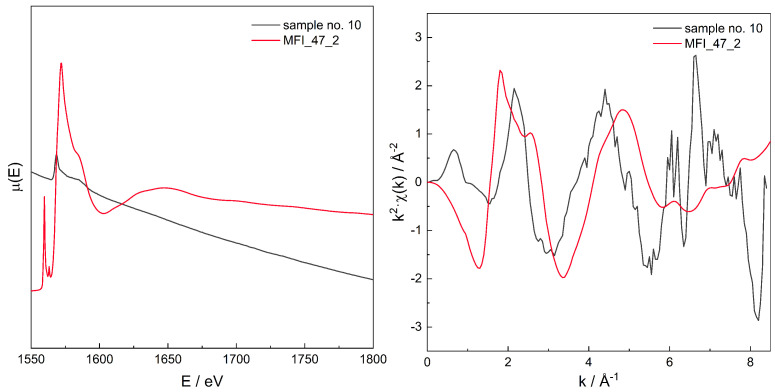

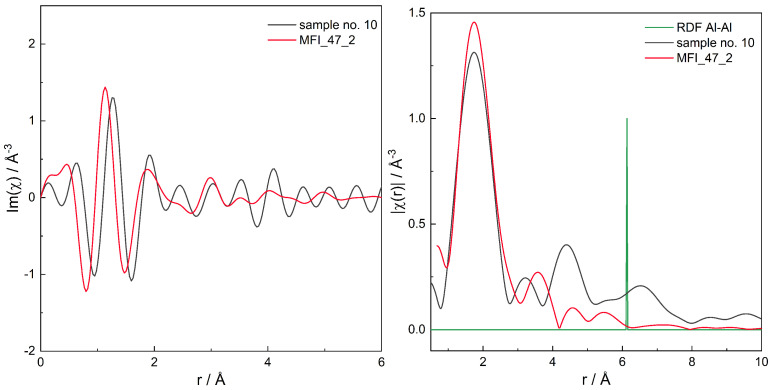
Spectrum of μ(E) (**upper left panel**), $k^{2}\chi \left(k\right)$ (**upper right**), ℑχ(r) (**lower left**), and $\left|\chi (r)\right|$ (**lower right**) for sample no. 10 (MFI of Si/Al = 25) and simulation for computational model labelled MFI_47_2. Matching RMS = 0.0658. The simulation model characterised by Al–Al distance of: 6.1 Å.

**Figure 12:**
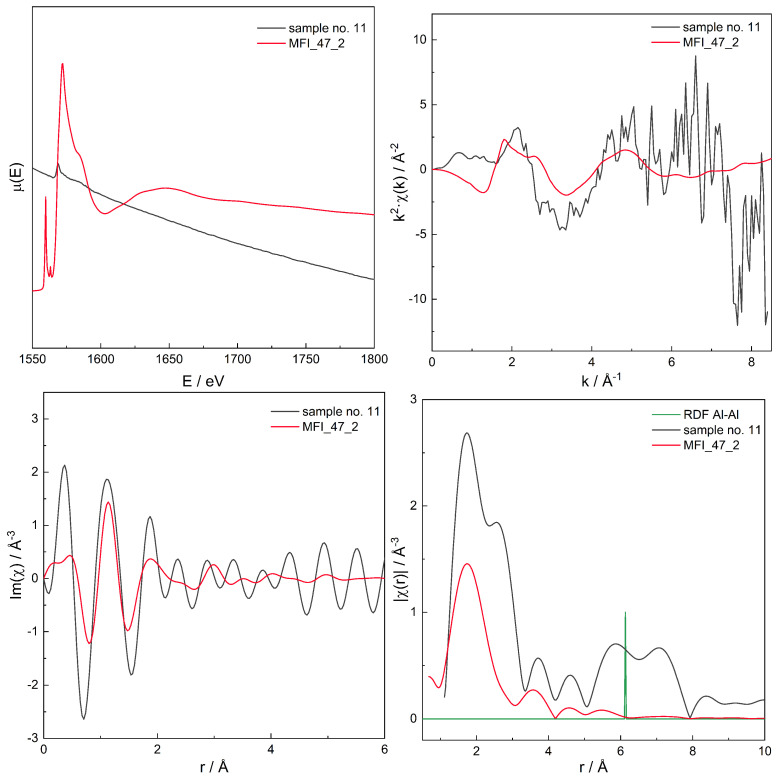
Spectrum of μ(E) (**upper left panel**), $k^{2}\chi \left(k\right)$ (**upper right**), ℑχ(r) (**lower left**), and $\left|\chi (r)\right|$ (**lower right**) for sample no. 11 (MFI of Si/Al = 40) and simulation for computational model labelled MFI_47_2. Matching RMS = 0.2010. The simulation model characterised by Al–Al distances of 6.1 Å.

For the series of models, for those with χ(r) modelled spectra best fitting to the experimental spectra for three zeolites with different Si/Al, the original, non-transformed spectra in the energy domain μ(E), the $k^{2}\chi \left(k\right)$, and the χ(r) spectra are presented in Figure 6, Figure 7, Figure 8, Figure 9, Figure 10, Figure 11 and Figure 12. For the latter, the matching RMS values are shown in the caption of each figure.

Due to the presence of the absorption edge of Si (1.8389 keV) relatively close to the Al edge (1.5596 keV), the EXAFS measurements were limited to the soft X-ray and hence to the moderate length of the wave vector *k*. Our study is concentrated on a few closest coordination spheres around emitters, hence for our purpose the central part of the *k*-spectrum (in the range of 2–6 Å^−1^) is important, and the spectrum beyond this range was damped by the Hanning function before the FT was obtained. The discrepancies for the shorter wave vector between experiment and simulations are caused by the build–up of the computational method inaccuracies along the flexible chains of the (rigid) SiO_4_ or AlO_4_ tetrahedra. Again, since our goal was to discriminate the similar structures of the same general framework and differing only by the Al substitution, we believe that the procedure is reliable.

The matching RMS in Figure 6 is very low for the tested model so the other Al distributions were not modelled.

The models have been constructed in a way so they have significant number of Al–O–Si–O–Al pairs or almost only isolated Al atoms. Thus, on the basis of RDF, for models which were positively verified by RMS, one can get another information on the Al distribution, based on the chosen range (for two Al separated by a single ${\left[{\mathrm{SiO}}_{4}\right]}^{4-}$ site the Al–Al distances are in the approximate range of 4.5–6.0 Å), with two ${\left[{\mathrm{SiO}}_{4}\right]}^{4-}$ sites (Al–Al distances of ca. 5.9–8 Å) or three ${\left[{\mathrm{SiO}}_{4}\right]}^{4-}$ sites (Al–Al distances of ca. 8–10 Å). Depending on the structure, the ranges can slightly overlap, though most of Al–Al distances are not close to the boundaries of the intervals/ranges and, thus, statistically significant information on Al distribution is delivered. In particular, models for samples 2 (FER) and 9 (MFI) have a significant number of Al–O–Si–O–Al pairs while Al atoms in the rest of the models are isolated.

#### Additivity of EXAFS Spectra

The EXAFS spectra are additive in *k*-space and, formally, they are not so in the reciprocal *R*-space, regardless of the linearity of the FT operator, which stems from the calculation of the amplitude (absolute value) of the complex FT. For the separated peaks, however, when their overlap as observed in the $\left|\chi (r)\right|$ plot is negligible (and hence their *ℜ* and *ℑ* do not cancel out), approximate additivity could be assumed. Nevertheless, for the purpose of the component analysis, the *k*-space spectra (in the range of 2–6 Å^−1^) were used.

The EXAFS signal χ(k) has been simulated for each non-equivalent Al position in the zeolite structure concerned (MFI, FER, MOR). Having components for each *T* position, the linear combinations have been calculated according to the Al distribution in models (Table 2) as a function:

(2) $$f_{\mathrm{comb}}=\sum_{i}c_{i}\cdot f_{Ti},$$

where $c_{i}$ are the coefficients (i⩾0) and $f_{Ti}$ are the EXAFS signals χ(k) for each non-equivalent Al position.

By performing the decomposition of the experimental results, one can estimate the contribution of each *T* position occupied by Al. The results of these fittings are gathered in Table 4. Although uncertainties are significant (as the spectral components are quite similar to each other), the numbers strongly indicate which of the crystallographic positions are mostly populated. It should not be, however, overlooked that the higher uncertainties can be caused by either the presence of Al positions other than in the standards used for decomposition, or by the geometry distortion (*vide supra*). Nonetheless, we believe that the procedure, even though of only qualitative or semi-quantitative accuracy, described in this article can serve as help when tackling the issue of solving systems of low symmetry and locally distorted geometry.

### 3.4. STEM

**Figure 13:**
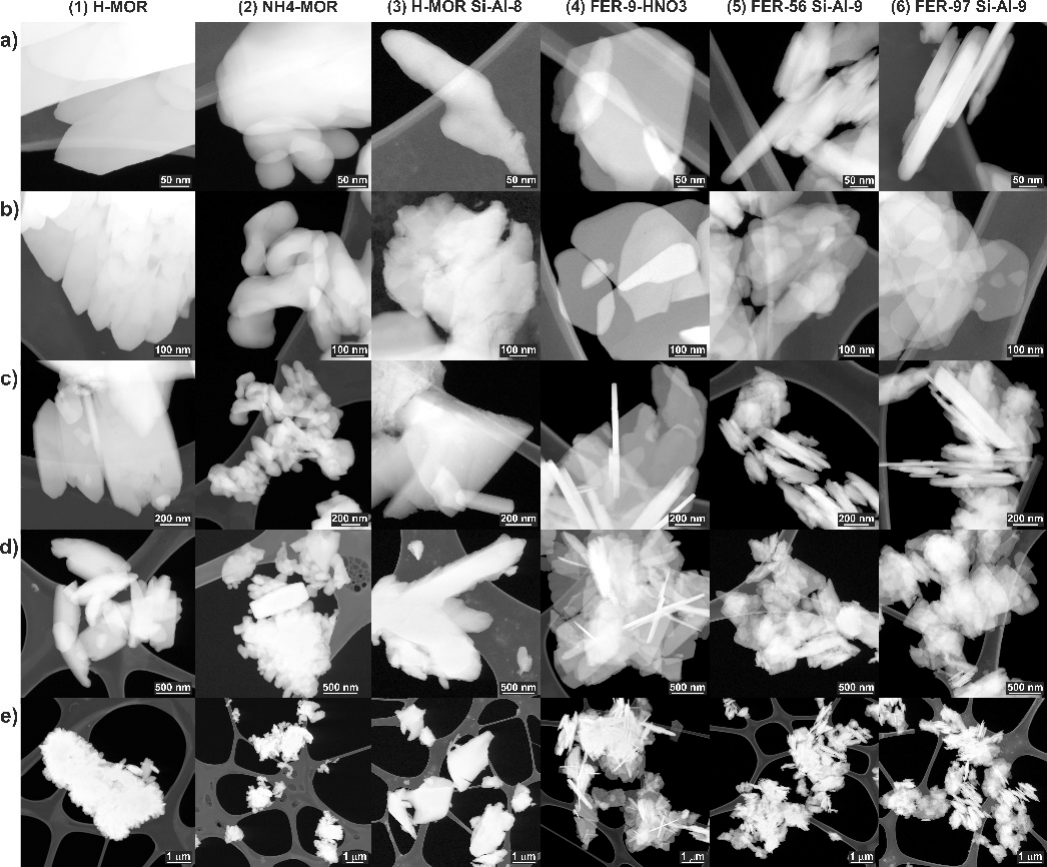
STEM overview and detail aspect images (**a**–**e**) of the zeolite morphology: mordenite (MOR, samples 1–3, columns 1–3, resp.), ferrierite (FER, samples 4–6, columns 4–6) samples of various Si/Al ratio.

Scanning Transmission Electron Microscopy (STEM) images were collected to assess the morphology of the prepared zeolites. STEM-HAADF micrographs (Figure 13) illustrate the distinct architecture of mordenite, ferrierite, faujasite, and beta zeolite. All investigated materials retained crystalline order. The mordenite and ferrierite zeolites present a bulk morphology with minor open microporosity (Figure 13 columns 1–6, resp.). The overview images of mordenite samples (Figure 13c–e, columns 1–3) show aggregated elongated nanocrystals of average length 500 nm to 1 m and 200 nm width. While the H-MORs show a more pronounced aspect ratio of the rod-shape nanocrystal bundles and chunks, the NH4-MOR reveals more oblique and refined nanocrystal morphology. Another three different ferrierite samples (Figure 13c–e, columns 4–6), respectively present very similar lamellar morphology of up to 1 m plane nanocrystals with very narrow 50–150 nm side dimensions. As presented in Figure 13 (FER, samples 4–6, columns 4–6), the morphology of ferrierite zeolites do not change significantly (in size and overall shape) under the influence of Si→Al exchange although a high content of Al is known to expand zeolite lattice constant.

## 4. Conclusions

The examined samples preserved their general structure regardless of the imposed Si/Al ratio and the samples high crystallinity.

As expected, the distribution of Al sites, although not strictly periodical, were not stochastic either. We conclude that the fully translational–symmetric periodic computational models, used in EXAFS simulations, can be regarded as the extreme cases of the real samples. In other words, the real samples are indeed the linear combinations of the idealised computational models.

Without the translational symmetric component of the Al distribution, it would be expected that the diffractograms would not have noticed the peak shifts and only peak broadening would have occurred—in such a case, only EXAFS (local geometry sensitive method, not dependent on the translational symmetry) would be able to observe the structural differences imposed by the Al introduction into the zeolite framework.

It was also found that the mere RDF function can be useful for the exclusion of the H-peaks from the calculated spectra for the purpose of the comparison thereof to the experimental spectra (calculation of the fitness).

It can be recalled here that the matching of the $\left|\chi (r)\right|$ results for the model and the samples allowed to indicate whether the Al pairs or rather the isolated Al atoms are preferred. 

## Figures and Tables

**Table 1:** A detailed description of the studied samples of zeolites. The value of $\Delta {\tilde{\nu }}_{\mathrm{OH}\cdots \mathrm{CO}}$ denotes the wavenumber shift of the OH band upon adsorption of CO. The $A_{330}/A_{170}$ entity is the ratio of absorbance measured at 330 °C with respect to the absorbance at 170 °C. The B.a.s (L.a.s) denotes the Brønsted (Lewis) acidic sites concentration in μmol/g.

Sample No.	Framework	Si/Al	Product ID	mfr.	B.a.s	L.a.s	(B + L) a.s.	$A_{330}/A_{170}$ (NH_3_)	$\Delta {\tilde{\nu }}_{\mathrm{OH}\cdots \mathrm{CO}}$/cm^−1^
1	MOR	15	660-HOA	Tosoh	1231	20	1251	0.9	313
2	MOR	10	CBV-21A	Tosoh	1635	5	1640	0.95	310
3	MOR	8	620-HOA	Tosoh	1790	10	1800	0.6	309
4	FER	9	— (own synthesis)		1100	0	1100	0.6	309
5	FER	9	— (own synthesis)		1363	51	1414	0.55	308
6	FER	9	— (own synthesis)		612	0	612	0.45	303
9	MFI	11.5	CBV-2314	Zeolyst					
10	MFI	25	CBV-5524G	Zeolyst					
11	MFI	40	CBV-8014	Zeolyst					
12	MFI	140	CBV-28014	Zeolyst					
13	MFI	750	HSZ-890HOA	Tosoh					

**Table 2:** Considered Al substitution positions in the FER, MFI, and MOR model unit cells.

Model Label	Framework	Si/Al	*T* Sites Substitutions
FER_8_1	FER	8	T1g, T3e, T4c, T4d
MFI_11_1	MFI	11.5	T7b, T7c, T7g, T10c, T10f, T10h, T12c, T12f
MFI_11_2	MFI	11.5	T1f, T6c, T7b, T7c, T7g, T10c, T10h, T12f
MFI_15_1	MFI	15	T8c, T9c, T10e, T12a, T12d, T12f
MFI_15_2	MFI	15	T8c, T9c, T10e, T11h, T12a, T12e
MFI_15_3	MFI	15	T8c, T9c, T10g, T11h, T12a, T12e
MFI_15_4	MFI	15	T6g, T9c, T10g, T11h, T12a, T12e
MFI_15_5	MFI	15	T8d, T9c, T9g, T11h, T12a, T12e
MFI_15_6	MFI	15	T9c, T9g, T11h, T12a, T12d, T12f
MFI_15_7	MFI	15	T3b, T9c, T9g, T11h, T12a, T12d
MFI_15_8	MFI	15	T6c, T6g, T9g, T11h, T12a, T12d
MFI_15_9	MFI	15	T3a, T6c, T6e, T6g, T9g, T11h
MFI_15_10	MFI	15	T3a, T3e, T6g, T8d, T9c, T9g
MFI_23_1	MFI	23	T10c, T10f, T12c, T12f
MFI_23_2	MFI	23	T1e, T10c, T12c, T12f
MFI_47_1	MFI	47	T12c, T12f
MFI_47_2	MFI	47	T3d, T12c
MOR_8_5	MOR	8.6	T2e, T3a, T3h, T4e, T4g
MOR_8_6	MOR	8.6	T2e, T4a, T4e, T4g, T4h
MOR_11_1	MOR	11	T2e, T3a, T3e, T3g
MOR_11_6	MOR	11	T2p, T3b, T3e, T3g
MOR_11_8	MOR	11	T2p, T3a, T3f, T3g
MOR_11_10	MOR	11	T2e, T3b, T3e, T4g
MOR_11_11	MOR	11	T1m, T2p, T3a, T4f
MOR_11_12	MOR	11	T1m, T2p, T4b, T4f
MOR_11_13	MOR	11	T1m, T1p, T2k, T2p
MOR_11_14	MOR	11	T2d, T2f, T3b, T4a
MOR_11_15	MOR	11	T1d, T2d, T2f, T3c
MOR_11_16	MOR	11	T1m, T2k, T2p, T3h
MOR_11_17	MOR	11	T2d, T2f, T3b, T3c
MOR_11_18	MOR	11	T2d, T2f, T2l, T3b
MOR_15_1	MOR	15	T2e, T3e, T3g
MOR_15_9	MOR	15	T2m, T3e, T3g

**Table 3:** Average nanograin size $D_{\mathrm{aver}}$ of the studied zeolite samples calculated from the Debye –Scherrer formula and the strain $\varepsilon_{\mathrm{UDSM}}$ calculated from the Williamson–Hall equation.

Sample No.	Framework	Si/Al	$D_{\mathrm{aver}}$/nm	=$\varepsilon_{\mathrm{UDSM}}\cdot 10^{3}$=
1	MOR	15	156±12	1.8
2	MOR	10	124±13	3.5
3	MOR	8	263±102	2.6
6	FER	9	47±1.4	1.8
9	MFI	11.5	77±1.5	4.5
10	MFI	25	460±67	2.3
11	MFI	40	222±19	2.6

**Table 4:** Values of the $c_{x}$ parameters calculated from the Equation (2) together with standard deviations and *t*-values (in parentheses). Dash (–) denotes that no satisfactory fit was found with concerned components.

Sample No.	Framework	Si/Al	T-Al Structure Contribution/%											
			$c_{1}$	$c_{2}$	$c_{3}$	$c_{4}$	$c_{5}$	$c_{6}$	$c_{7}$	$c_{8}$	$c_{9}$	$c_{10}$	$c_{11}$	$c_{12}$
1	MOR	15	0 ± 0.40 (0)	0 ± 0.43 (0)	1.15 ± 0.67 (1.70)	0 ± 0.69 (0)								
2	MOR	10	0 ± 0.44 (0)	0 ± 0.48 (0)	0.84 ± 0.74 (1.13)	0 ± 0.78 (0)								
3	MOR	8	0 ± 0.60 (0)	0 ± 0.64 (0)	1.91 ± 1.00 (1.83)	0 ± 1.04 (0)								
4	FER	9	0.14 ± 0.63 (0)	0 ± 0.65 (0)	0.55 ± 0.40 (1.38)	0 ± 0.79 (0)								
5	FER	9	0 ± 0.76 (0)	0 ± 0.79 (0)	0.59 ± 0.48 (1.20)	0 ± 0.96 (0)								
6	FER	9	0.24 ± 0.97 (0.24)	0 ± 1.02 (0)	0.87 ± 0.62 (1.41)	0 ± 1.22 (0)								
9	MFI	11.5	2.35 ± 3.17 (0.74)	0 ± 6.18 (0)	0 ± 5.81 (0)	0 ± 6.24 (0)	0 ± 6.69 (0)	0 ± 6.22 (0)	0 ± 5.64 (0)	0 ± 5.64 (0)	0 ± 7.13 (0)	0 ± 4.92 (0)	0 ± 9.84 (0)	0 ± 6.84 (0)
10	MFI	25	0.54 ± 1.03 (0.53)	0 ± 2.00 (0)	0 ± 1.88 (0)	0 ± 2.02 (0)	0 ± 2.16 (0)	0 ± 2.01 (0)	0 ± 1.82 (0)	0 ± 1.82 (0)	0 ± 2.31 (0)	0 ± 1.59 (0)	0 ± 3.18 (0)	0 ± 2.21 (0)
11	MFI	40	2.00 ± 1.38 (1.45)	0 ± 2.69 (0)	0 ± 2.53 (0)	0 ± 2.72 (0)	0.07 ± 2.91 (0.02)	0 ± 2.71 (0)	0 ± 2.46 (0)	0 ± 2.46 (0)	0 ± 3.11 (0)	0 ± 2.15 (0)	0 ± 4.29 (0)	0 ± 2.98 (0)

## Data Availability

The data presented in this study are available in Supplementary Material.

## Supplementary Materials

The following are available online. Figure S1: Differential diffractograms of silicalite (IZA), studied samples, and computational models for MFI, MOR, FER, Figure S2: PXRD diffractograms of silicalite (IZA), silicalite (DFT), and computational model for MFI, Figure S3: Spectrum of $k^{2}\chi \left(k\right)$ for sum of non-equivalent Al position observed in model and simulation for computational model labelled FER_8_1, Figure S4: Spectrum of $k^{2}\chi \left(k\right)$ for sum of non-equivalent Al position observed in model and simulation for computational model labelled MOR_11_10, Figure S5: Spectrum of $k^{2}\chi \left(k\right)$ for sum of non-equivalent Al position observed in model and simulation for computational model labelled MOR_11_15, Figure S6: Spectrum of $k^{2}\chi \left(k\right)$ for sum of non-equivalent Al position observed in model and simulation for computational model labelled MOR_8_6, Figure S7: Spectrum of $k^{2}\chi \left(k\right)$ for sum of non-equivalent Al position observed in model and simulation for computational model labelled MFI_11_2, Figure S8: Spectrum of $k^{2}\chi \left(k\right)$ for sum of non-equivalent Al position observed in model and simulation for computational model labelled MFI_47_2.

## Sample Availability

Samples of the compounds are available from the authors.

## References

[1] Strzempek M., Tarach K.A., Góra-Marek K., Rey F., Palomino M., Valencia S., Piskorz W. (2021). Multiscale exploration of hydrocarbon adsorption and hopping through ZSM-5 channels—From Monte Carlo modelling to experiment. Phys. Chem. Chem. Phys..

[2] Vermeiren W., Gilson J.P. (2009). Impact of Zeolites on the Petroleum and Petrochemical Industry. Top. Catal..

[3] Guisnet M., Gilson J.P. (2002). Zeolites for Cleaner Technologies.

[4] Corma A., Martínez A., Čejka J., van Bekkum H. (2005). Zeolites in refining and petrochemistry. Zeolites and Ordered Mesoporous Materials: Progress and Prospects.

[5] Olsbye U., Svelle S., Bjørgen M., Beato P., Janssens T.V.W., Joensen F., Bordiga S., Lillerud K.P. (2012). Conversion of methanol to hydrocarbons: How zeolite cavity and pore size controls product selectivity. Angew. Chem. Int. Ed..

[6] Olah G.A. (2013). Towards Oil Independence Through Renewable Methanol Chemistry. Angew. Chem. Int. Ed..

[7] Liebau F., Gies H., Gunawardane R.P., Marler B. (1986). Classification of tectosilicates and systematic nomenclature of clathrate type tectosilicates: A proposal. Zeolites.

[8] Loewenstein W. (1954). The distribution of aluminum in the tetrahedra of silicates and aluminates. Am. Mineral..

[9] Dempsey E., Kuehl G.H., Olson D.H. (1969). Variation of the lattice parameter with aluminum content in synthetic sodium faujasites. Evidence for ordering of the framework ions. J. Phys. Chem..

[10] Tielens F., Langenaeker W., Geerlings P. (2000). Ab initio study of the bridging hydroxyl acidity and stability in the 12-membered ring of zeolites. J. Mol. Struct. THEOCHEM.

[11] Piskorz W., Zasada F., Broclawik E., Borowski T., Radoń M. (2019). Catalytic Properties of Selected Transition Metal Oxides-Computational Studies. Transition Metals in Coordination Environments. Computational Chemistry and Catalysis Viewpoints.

[12] Kornatowski J., Sychev M., Kuzenkov S., Strnadová K., Pilz W., Kassner D., Pieper G., Baur W.H. (1995). V-Ti and V-Al silicate molecular sieves of MFI topology: Synthesis and characteristics. J. Chem. Soc. Faraday Trans..

[13] Kowalak S., Stawiński K., Makowiak A. (2001). Incorporation of zinc into silica mesoporous molecular sieves. Microporous Mesoporous Mater..

[14] Nogier J.P., Millot Y., Man P.P., Shishido T., Che M., Dzwigaj S. (2009). Probing the incorporation of Ti(IV) into the BEA zeolite framework by XRD, FTIR, NMR, and DR UV-jp810722bis. J. Phys. Chem. C.

[15] Trejda M., Ziolek M., Millot Y., Chalupka K., Che M., Dzwigaj S. (2011). Methanol oxidation on VSiBEA zeolites: Influence of V content on the catalytic properties. J. Catal..

[16] Xu R., Pang W., Yu J., Huo Q., Chen J. (2007). Chemistry of Zeolites and Related Porous Materials Synthesis and Structure.

[17] Meeprasert J., Kungwan N., Jungsuttiwong S., Namuangruk S. (2014). Location and reactivity of extra-framework cation in the alkali exchanged LTL zeolites: A periodic density functional study. Microporous Mesoporous Mater..

[18] Tielens F., Dzwigaj S. (2010). Group V metal substitution in silicate model zeolites: In search for the active site. Chem. Phys. Lett..

[19] Kozyra P., Piskorz W. (2016). A comparative computational study on hydrogen adsorption on the Ag^+^, Cu^+^, Mg^2+^, Cd^2+^, and Zn^2+^ cationic sites in zeolites. Phys. Chem. Chem. Phys..

[20] Woertink J.S., Smeets P.J., Groothaert M.H., Vance M.A., Sels B.F., Schoonheydt R.A., Solomon E.I. (2009). A [Cu2O]^2+^ core in Cu-ZSM-5, the active site in the oxidation of methane to methanol. Proc. Natl. Acad. Sci. USA.

[21] Selvaraj T., Rajalingam R., Balasubramanian V. (2018). Impact of zeolite-Y framework on the geometry and reactivity of Ru (III) benzimidazole complexes—A DFT study. Appl. Surf. Sci..

[22] IZA-SC Database of Zeolite Structures. Zeolite Framework Types. https://europe.iza-structure.org/IZA-SC/ftc_table.php.

[23] Bhan A., Iglesia E. (2008). A Link between Reactivity and Local Structure in Acid Catalysis on Zeolites. Accounts Chem. Res..

[24] Mlekodaj K., Dedecek J., Pashkova V., Tabor E., Klein P., Urbanova M., Karcz R., Sazama P., Whittleton S.R., Thomas H.M. (2018). Al organization in the SSZ-13 Zeolite. Al distribution and extraframework sites of divalent cations. J. Phys. Chem. C.

[25] Akporiaye D.E., Dahl I.M., Mostad H.B., Wendelbo R. (1996). Aluminum distribution in chabazite: An experimental and computational study. J. Phys. Chem..

[26] Benco L., Bucko T., Hafner J., Toulhoat H. (2005). Periodic DFT calculations of the stability of Al/Si substitutions and extraframework Zn^2+^ cations in mordenite and reaction pathway for the dissociation of H2 and CH4. J. Phys. Chem. B.

[27] García-Pérez E., Dubbeldam D., Liu B., Smit B., Calero S. (2007). A computational method to characterize framework aluminum in aluminosilicates. Angew. Chem. Int. Ed..

[28] Olson D.H., Khosrovani N., Peters A.W., Toby B.H. (2000). Crystal structure of dehydrated CsZSM-5 (5.8Al): Evidence for nonrandom aluminum distribution. J. Phys. Chem. B.

[29] Lu B., Kanai T., Oumi Y., Sano T. (2007). Aluminum distribution in high-silica mordenite. J. Porous Mater..

[30] Sazama P., Tabor E., Klein P., Wichterlova B., Sklenak S., Mokrzycki L., Pashkova V., Ogura M., Dedecek J. (2016). Al-rich beta zeolites. Distribution of Al atoms in the framework and related protonic and metal-ion species. J. Catal..

[31] Di Iorio J.R., Gounder R. (2016). Controlling the Isolation and Pairing of Aluminum in Chabazite Zeolites Using Mixtures of Organic and Inorganic Structure-Directing Agents. Chem. Mater..

[32] Klinowski J. (1984). Nuclear magnetic resonance studies of zeolites. Prog. Nucl. Magn. Reson. Spectrosc..

[33] Majda D., Paz F.A., Friedrichs D., Foster M.D., Simperler A., Bell R.G., Klinowski J. (2008). Hypothetical zeolitic frameworks: In search of potential heterogeneous catalysts. J. Phys. Chem. C.

[34] Porcher F.F., Souhassou M., Lecomte C.E.P. (2014). Experimental determination of electrostatic properties of Na-X zeolite from high resolution X-ray diffraction. Phys. Chem. Chem. Phys..

[35] Lesthaeghe D., Horré A., Waroquier M., Marin G.B., Van Speybroeck V. (2009). Theoretical Insights on Methylbenzene Side-Chain Growth in ZSM-5 Zeolites for Methanol-to-Olefin Conversion. Chem. A Eur. J..

[36] Joyner R.W., Smith A.D., Stockenhuber M., van den Berg M.W.E. (2004). The local structure of aluminium sites in zeolites. Phys. Chem. Chem. Phys..

[37] Joyner R.W., Smith A.D., Stockenhuber M., van den Berg M.W.E. (2004). A soft X-ray exafs study of the local structure of tetrahedral aluminium in zeolites. Stud. Surf. Sci. Catal..

[38] Kulkarni A.R., Zhao Z.J., Siahrostami S., Nørskov J.K., Studt F. (2018). Cation-exchanged zeolites for the selective oxidation of methane to methanol. Catal. Sci. Technol..

[39] Iwamoto M., Yokoo S., Sakai K., Kagawa S. (1981). Catalytic decomposition of nitric oxide over copper(II)-exchanged, Y-type zeolites. J. Chem. Soc. Faraday Trans. 1.

[40] Iwamoto M., Furukawa H., Mine Y., Uemura F., Mikuriya S.i., Kagawa S. (1986). Copper(II) ion-exchanged ZSM-5 zeolites as highly active catalysts for direct and continuous decomposition of nitrogen monoxide. J. Chem. Soc. Chem. Commun..

[41] Oda A., Torigoe H., Itadani A., Ohkubo T., Yumura T., Kobayashi H., Kuroda Y. (2012). Unprecedented reversible redox process in the ZnMFI-H2 system involving formation of stable atomic Zn0. Angew. Chem. Int. Ed..

[42] Dubkov K., Ovanesyan N., Shteinman A., Starokon E., Panov G. (2002). Evolution of Iron States and Formation of *α*-Sites upon Activation of FeZSM-5 Zeolites. J. Catal..

[43] Starokon E.V., Parfenov M.V., Arzumanov S.S., Pirutko L.V., Stepanov A.G., Panov G.I. (2013). Oxidation of methane to methanol on the surface of FeZSM-5 zeolite. J. Catal..

[44] Xu J., Armstrong R.D., Shaw G., Dummer N.F., Freakley S.J., Taylor S.H., Hutchings G.J. (2016). Continuous selective oxidation of methane to methanol over Cu- and Fe-modified ZSM-5 catalysts in a flow reactor. Catal. Today.

[45] Park K.S., Kim J.H., Park S.H., Moon D.J., Roh H.S., Chung C.H., Um S.H., Choi J.H., Bae J.W. (2017). Direct activation of CH4 to oxygenates and unsaturated hydrocarbons using N_2_O on Fe-modified zeolites. J. Mol. Catal. A Chem..

[46] Stepanov A.G., Arzumanov S.S., Gabrienko A.A., Parmon V.N., Ivanova I.I., Freude D. (2008). Significant influence of Zn on activation of the C-H bonds of small alkanes by brønsted acid sites of zeolite. ChemPhysChem.

[47] Biscardi J.A., Meitzner G.D., Iglesia E. (1998). Structure and density of active Zn species in Zn/H-ZSM5 propane aromatization catalysts. J. Catal..

[48] Niu X., Gao J., Miao Q., Dong M., Wang G., Fan W., Qin Z., Wang J. (2014). Influence of preparation method on the performance of Zn-containing HZSM-5 catalysts in methanol-to-aromatics. Microporous Mesoporous Mater..

[49] Wang X., Xu J., Qi G., Li B., Wang C., Deng F. (2013). Alkylation of benzene with methane over ZnZSM-5 zeolites studied with solid-state NMR spectroscopy. J. Phys. Chem. C.

[50] Smeets P.J., Woertink J.S., Sels B.F., Solomon E.I., Schoonheydt R.A. (2010). Transition-metal ions in zeolites: Coordination and activation of oxygen. Inorg. Chem..

[51] Pietrzyk P., Piskorz W., Sojka Z., Broclawik E. (2003). Molecular structure, spin density distribution, and hyperfine coupling constants of the *η*1{CuNO}11 adduct in the ZSM-5 zeolite: DFT calculations and comparison with EPR data. J. Phys. Chem. B.

[52] Uzunova E.L., Göltl F., Kresse G., Hafner J. (2009). Application of hybrid functionals to the modeling of NO adsorption on Cu-SAPO-34 and Co-SAPO-34: A periodic DFT study. J. Phys. Chem. C.

[53] Davidová M., Nachtigallová D., Nachtigall P., Sauer J. (2004). Nature of the Cu+-NO bond in the gas phase and at different types of Cu+ sites in zeolite catalysts. J. Phys. Chem. B.

[54] Izquierdo R., Rodríguez L.J., Añez R., Sierraalta A. (2011). Direct catalytic decomposition of NO with Cu-ZSM-5: A DFT-ONIOM study. J. Mol. Catal. A Chem..

[55] Göltl F., Hafner J. (2012). Structure and properties of metal-exchanged zeolites studied using gradient-corrected and hybrid functionals. III. Energetics and vibrational spectroscopy of adsorbates. J. Chem. Phys..

[56] Heyden A., Peters B., Bell A.T., Keil F.J. (2005). Comprehensive DFT study of nitrous oxide decomposition over Fe-ZSM-5. J. Phys. Chem. B.

[57] Pietrzyk P., Sojka Z., Dzwigaj S., Che M. (2007). Generation, identification, and reactivity of paramagnetic VO2 centers in zeolite BEA for model studies of processes involving spin pairing, electron transfer, and oxygen transfer. J. Am. Chem. Soc..

[58] Behrens P., Karge H.G., Weitkamp J. (2004). XANES, EXAFS and Related Techniques. Molecular Sieves—Science and Technology. Characterization I.

[59] Van Bokhoven J.A., van der Eerden A.M.J., Koningsberger D.C. (2003). Three-Coordinate Aluminum in Zeolites Observed with In situ X-ray Absorption Near-Edge Spectroscopy at the Al K-Edge: Flexibility of Aluminum Coordinations in Zeolites. J. Am. Chem. Soc..

[60] Bianconi A., Kanamori J., Kotani A. (1988). One-Electron Transitions in the XANES of Condensed Systems. Core-Level Spectroscopy in Condensed Systems.

[61] Newville M. (2001). EXAFS analysis using FEFF and FEFFIT. J. Synchrotron Radiat..

[62] Bugaev L.A., van Bokhoven J.A., Sokolenko A.P., Latokha Y.V., Avakyan L.A. (2005). Local Structure of Aluminum in Zeolite Mordenite as Affected by Temperature. J. Phys. Chem. B.

[63] Zaja̧c M., Giela T., Freindl K., Kollbek K., Korecki J., Madej E., Pitala K., Kozioł-Rachwał A., Sikora M., Spiridis N. (2021). The first experimental results from the 04BM (PEEM/XAS) beamline at Solaris. Nucl. Instruments Methods Phys. Res. Sect. B Beam Interact. Mater. Atoms.

[64] Lutterotti L., Bortolotti M., Ischia G., Lonardelli I., Wenk H.R. (2007). Rietveld texture analysis from diffraction images. Z. Krist. Suppl..

[65] Lutterotti L., Pillière H., Fontugne C., Boullay P., Chateigner D. (2019). Full-profile search–match by the Rietveld method. J. Appl. Crystallogr..

[66] Kresse G., Furthmüller J. (1996). Efficiency of ab-initio total energy calculations for metals and semiconductors using a plane-wave basis set. Comput. Mater. Sci..

[67] Kresse G., Hafner J. (1993). Ab initio molecular dynamics for open-shell transition metals. Phys. Rev. B.

[68] Blöchl P.E. (1994). Projector augmented-wave method. Phys. Rev. B.

[69] Kresse G., Joubert D. (1999). From ultrasoft pseudopotentials to the projector augmented-wave method. Phys. Rev. B.

[70] Methfessel M.S., Paxton A.T. (1989). High-precision sampling for Brillouin-zone integration in metals. Phys. Rev. B.

[71] Perdew J.P., Burke K., Ernzerhof M. (1996). Generalized gradient approximation made simple. Phys. Rev. Lett..

[72] Perdew J.P., Burke K., Ernzerhof M. (1997). Erratum to Generalized Gradient Approximation Made Simple. Phys. Rev. Lett..

[73] Grimme S. (2004). Accurate description of van der Waals complexes by density functional theory including empirical corrections. J. Comput. Chem..

[74] Murnaghan F.D. (1944). The compressibility of media under extreme pressures. Proc. Natl. Acad. Sci. USA.

[75] Cabaret D., Sainctavit P., Ildefonse P., Flank A.M. (1996). Full multiple-scattering calculations on silicates and oxides at the Al K edge. J. Phys. Condens. Matter.

[76] Van Bokhoven J.A., Nabi T., Sambe H., Ramaker D.E., Koningsberger D.C. (2001). Interpretation of the Al K- and L II/III -edges of aluminium oxides: Differences between tetrahedral and octahedral Al explained by different local symmetries. J. Phys. Condens. Matter.

[77] Joly Y. (2001). X-ray absorption near-edge structure calculations beyond the muffin-tin approximation. Phys. Rev. B.

[78] Guda S.A., Guda A.A., Soldatov M.A., Lomachenko K.A., Bugaev A.L., Lamberti C., Gawelda W., Bressler C., Smolentsev G., Soldatov A.V. (2015). Optimized Finite Difference Method for the Full-Potential XANES Simulations: Application to Molecular Adsorption Geometries in MOFs and Metal–Ligand Intersystem Crossing Transients. J. Chem. Theory Comput..

[79] Bourke J.D., Chantler C.T., Joly Y. (2016). FDMX: Extended X-ray absorption fine structure calculations using the finite difference method. J. Synchrotron Radiat..

[80] Ravel B., Newville M. (2005). ATHENA, ARTEMIS, HEPHAESTUS: Data analysis for X-ray absorption spectroscopy using IFEFFIT. J. Synchrotron Radiat..

[81] Ravel B. (2018). Demeter: XAS Data Processing and Analysis. https://bruceravel.github.io/demeter/.

[82] Mote V.D., Purushotham Y., Dole B.N. (2012). Williamson-Hall analysis in estimation of lattice strain in nanometer-sized ZnO particles. J. Theor. Appl. Phys..

[83] Dubbeldam D., Torres-Knoop A., Walton K.S. (2013). On the inner workings of Monte Carlo codes. Mol. Simul..

[84] Dubbeldam D., Calero S., Ellis D.E., Snurr R.Q. (2016). RASPA: Molecular simulation software for adsorption and diffusion in flexible nanoporous materials. Mol. Simul..

[85] Pellicer-Porres J., Segura A., Martínez-Criado G., Rodríguez-Mendoza U.R., Lavín V. (2013). Formation of nanostructures in Eu^3+^ doped glass–ceramics: An XAS study. J. Phys. Condens. Matter.

