# Supplementary material for: Structural Studies of Aluminated form of Zeolites—EXAFS and XRD Experiment, STEM Micrography, and DFT Modelling
Source: Molecules. 2021 Jun 10;26(12):3566. doi: 10.3390/molecules26123566 (PMC8230598; doi:10.3390/molecules26123566)
Supplement: Supplementary file 1 [file molecules-26-03566-s001.zip › molecules-1205048-supplementary.pdf]

Article

Structural Studies of Aluminated Form of Zeolites–EXAFS Experiment and DFT Modelling. Supplementary information

Gabriela Jajko[1], Paweł Kozyra[1], Maciej Strzempek[1], Paulina Indyka[1,2], Marcin Zając[3], Stefan Witkowski[1], Witold Piskorz[1]*

[1]Faculty of Chemistry, Jagiellonian University in Kraków, ul. Gronostajowa 2, 30-387 Kraków, Poland
[2]Małopolska Centre of Biotechnology, ul. Gronostajowa 7A, 30-387 Kraków, Poland
[3]National Synchrotron Radiation Centre SOLARIS, Jagiellonian University in Kraków, ul. Czerwone Maki 98, 30-389 Kraków, Poland
* corresponding author: wpiskorz@chemia.uj.edu.pl, tel.: +48 12 686 2496.

Citation: Structural Studies of Aluminated Form of Zeolites–EXAFS Experiment and DFT Modelling. Supplementary information. *Molecules* **2021**, *26*, 3566. https://doi.org/10.3390/molecules26123566

Received: 15 April 2021
Accepted: 7 June 2021
Published: 10 June 2021

Publisher's Note: MDPI stays neutral with regard to jurisdictional claims in published maps and institutional affiliations.

Copyright: © 2021 by the authors. Submitted to *Molecules* for possible open access publication under the terms and conditions of the Creative Commons Attribution (CC BY) license (https://creativecommons.org/licenses/by/ 4.0/).

In the Supplementary Information section we provide the details of the diffractograms and the $k^2\chi(k)$ spectra for the sum of non-equivalent Al position observed in the model and the simulation for the computational models.

XRD

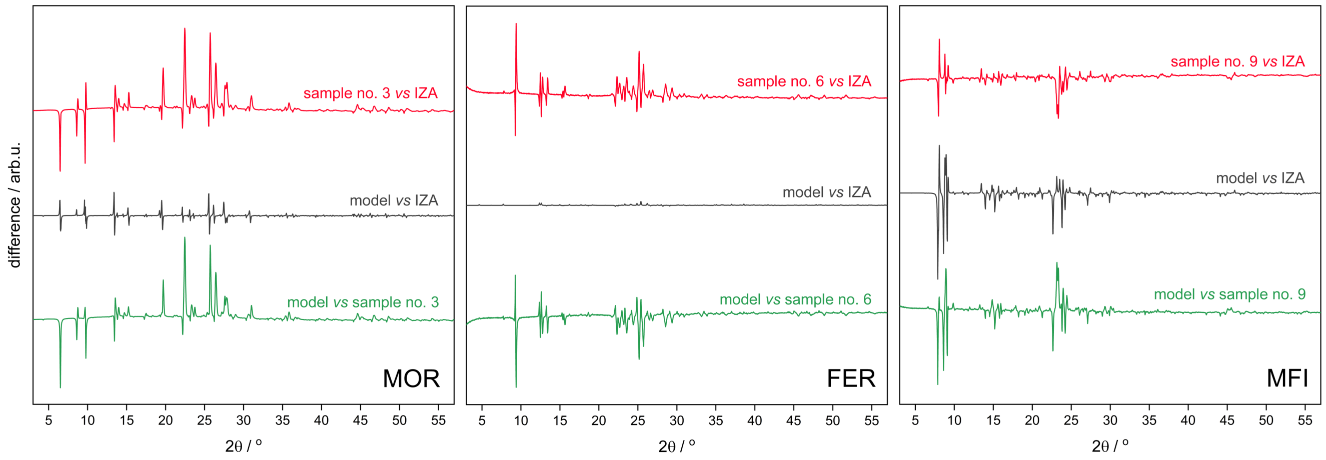

Figure S1. Differential diffractograms of silicalite (IZA), studied samples, and computational models for MFI, MOR, FER.

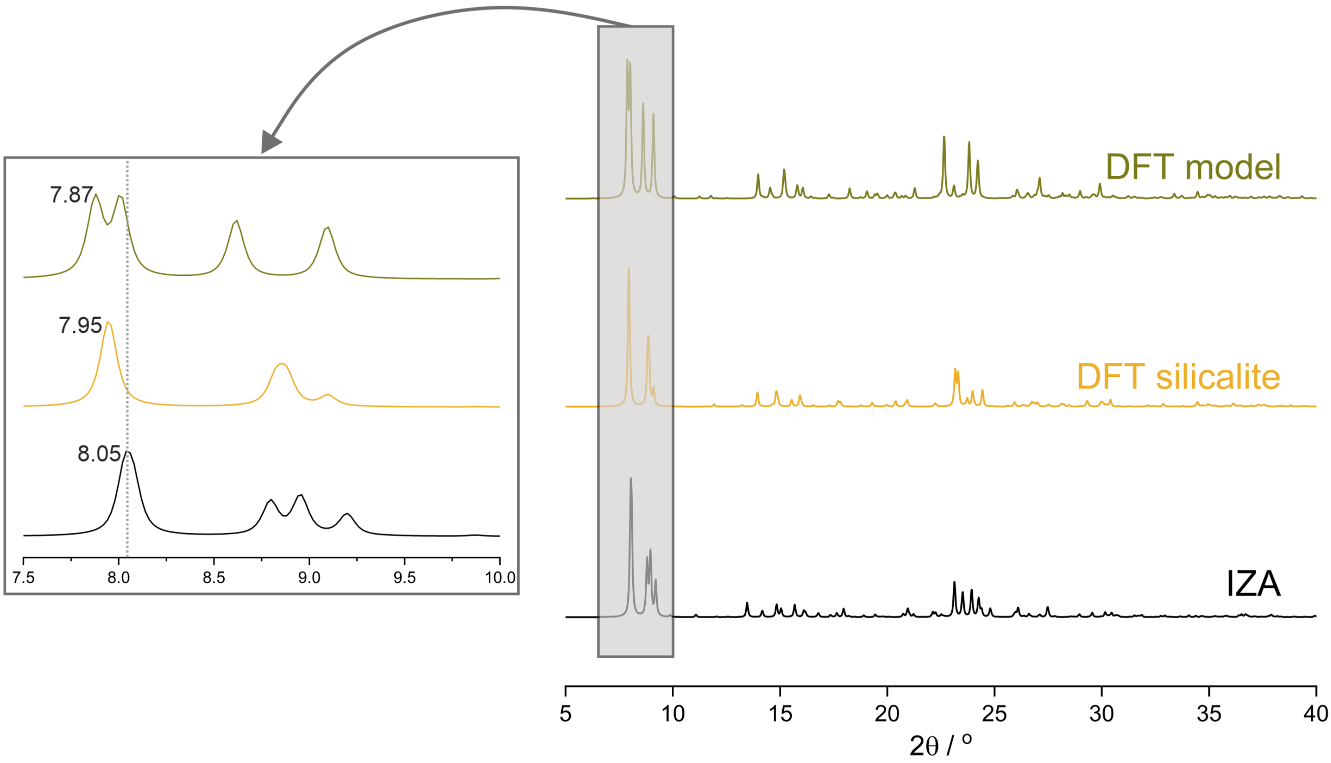

Figure S2. PXRD diffractograms of silicalite (IZA), silicalite (DFT), and computational model for MFI.

EXAFS

The results of EXAFS signal $|\chi(r)|$ for each non-equivalent Al position comparison with those obtained for the models.

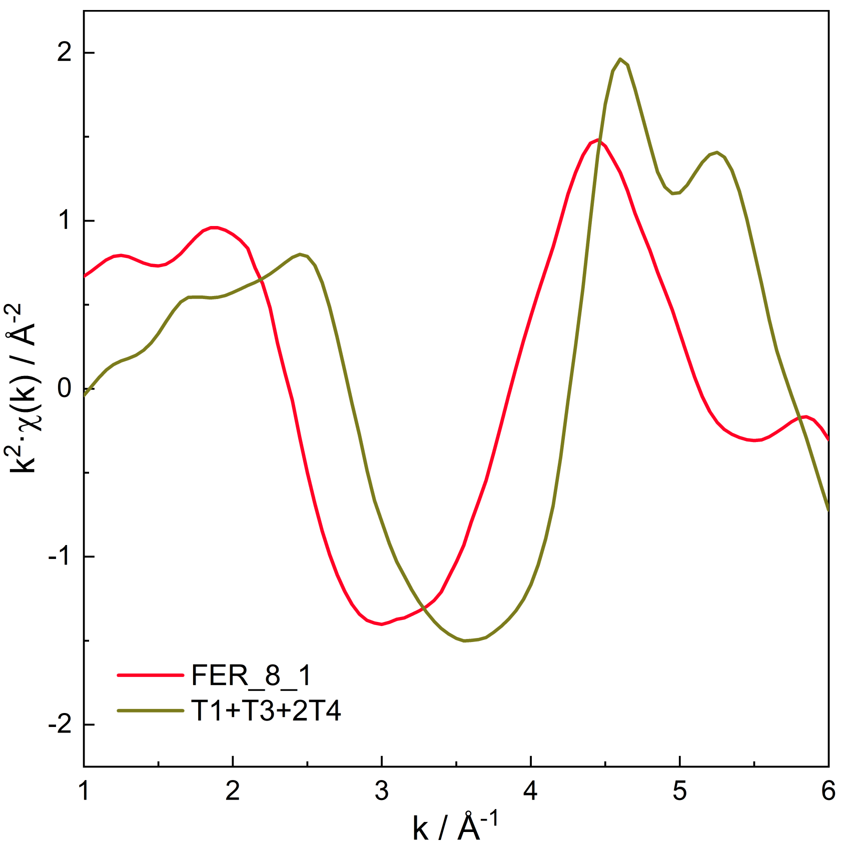

Figure S3. Spectrum of $k^2\chi(k)$ for sum of non-equivalent Al position observed in model and simulation for computational model labelled FER_8_1.

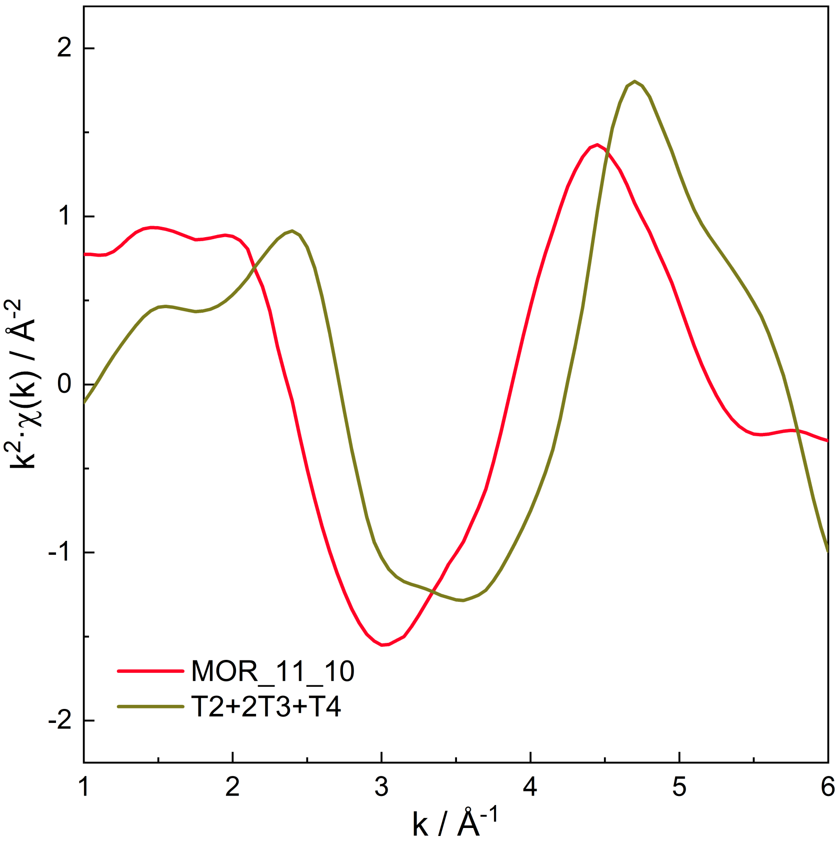

Figure S4. Spectrum of $k^2\chi(k)$ for sum of non-equivalent Al position observed in model and simulation for computational model labelled MOR_11_10.

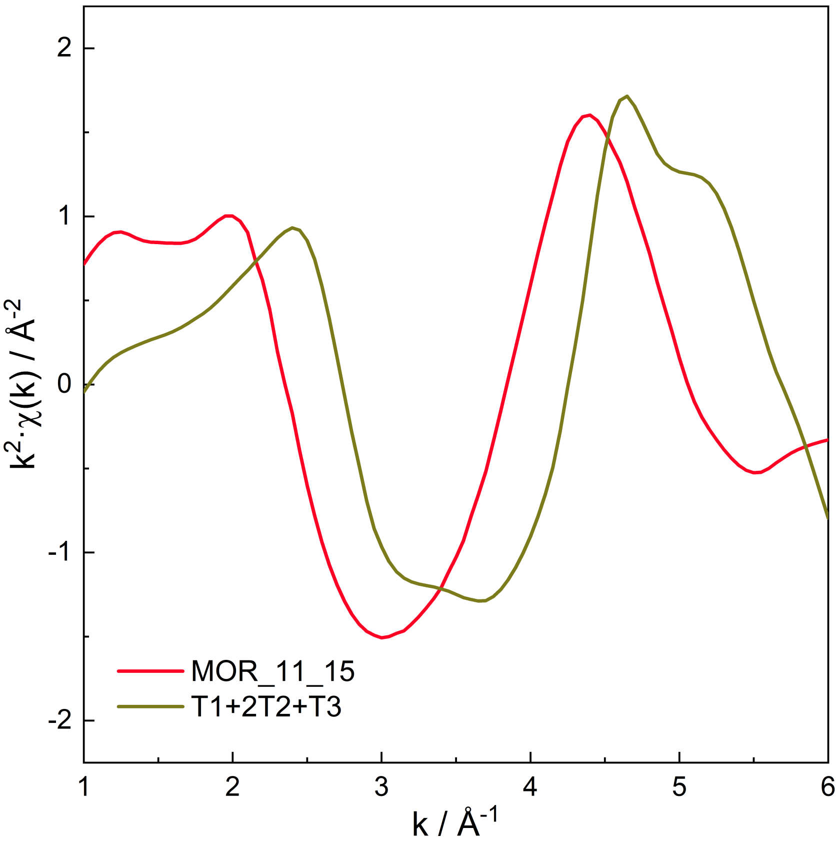

Figure S5. Spectrum of $k^2\chi(k)$ for sum of non-equivalent Al position observed in model and simulation for computational model labelled MOR_11_15.

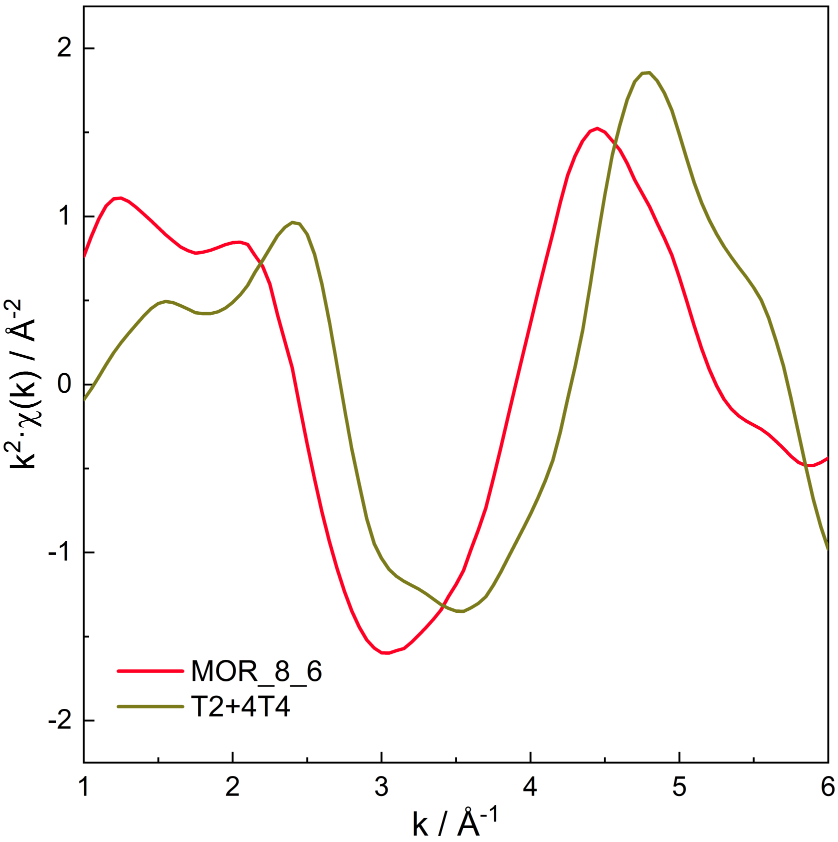

Figure S6. Spectrum of $k^2\chi(k)$ for sum of non-equivalent Al position observed in model and simulation for computational model labelled MOR_8_6.

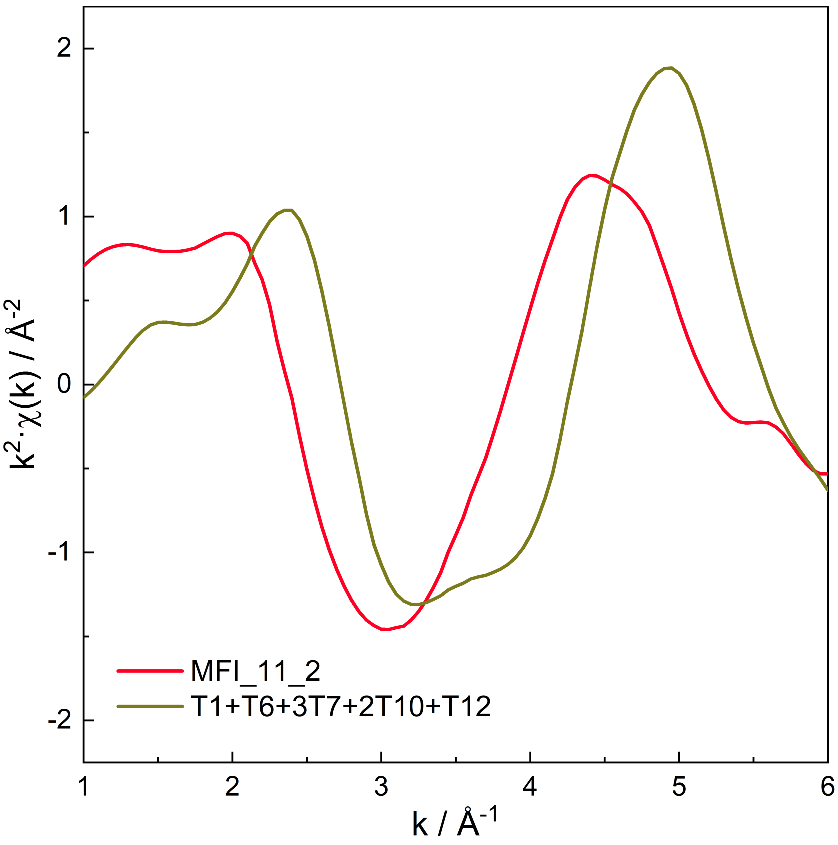

Figure S7. Spectrum of $k^2\chi(k)$ for sum of non-equivalent Al position observed in model and simulation for computational model labelled MFI_11_2.

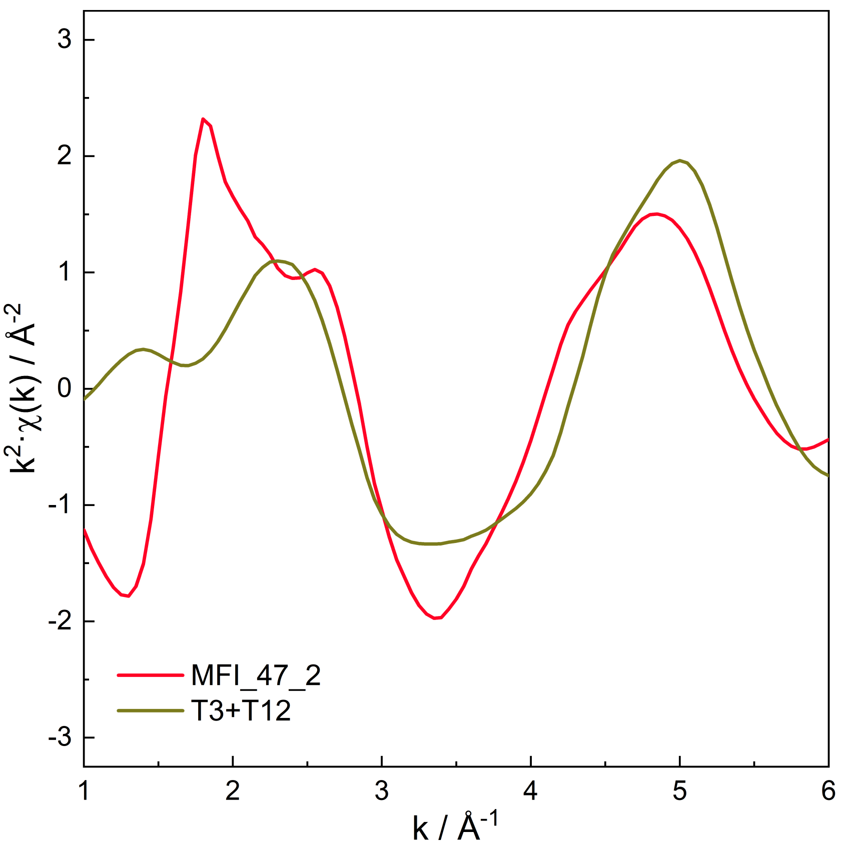

Figure S8. Spectrum of $k^2\chi(k)$ for sum of non-equivalent Al position observed in model and simulation for computational model labelled MFI_47_2.
